# The Patterns of Male and Female Flowers in Flowering Stage May Not Be Optimal Resource Allocation for Fruit and Seed Growth

**DOI:** 10.3390/plants10122819

**Published:** 2021-12-20

**Authors:** Lei Gao, Guozhu Yu, Fangyu Hu, Zhiqi Li, Weihua Li, Changlian Peng

**Affiliations:** Guangdong Provincial Key Laboratory of Biotechnology for Plant Development, School of Life Sciences, South China Normal University, No. 55 Zhongshan Avenue West, Tianhe District, Guangzhou 510631, China; ygz505164025@163.com (G.Y.); HUFANGYU1998@163.com (F.H.); gdflzqgg0@163.com (Z.L.); whli@scnu.edu.cn (W.L.); pengchl@scib.ac.cn (C.P.)

**Keywords:** *Cucumber sativus*, plant fitness, sex differentiation, monoecious, pollen resource

## Abstract

Changes in the proportions of male and female flowers in monoecious plants in response to external environmental conditions are directly related to the reproductive fitness of plants. The monoecious cucumber (*Cucumber sativus*) plant was used in this study to assess the responses of sex differentiation and the breeding process to nutrient supply and the degree of artificial pollination using pollen solutions of different concentrations. We found that the nutrient supply significantly improved the number of female flowers, while pollination treatments did not obviously increase the number of male flowers. Continuous pollination changed the number of female flowers especially in the later stage of the pollination experiment. Therefore, pollination changed the ratio of male and female flowers in the flowering stage of cucumber. Pollination treatment affected the fruit growth, seed set, and fruit yield. The number of fruit, fruit set percentage, and total seeds per plant did not increase with the pollination level, but individual fruit weight and seed number in one fruit did increase. The differentiation of male and female flowers in the flowering stage of cucumber is a response to nutrient and pollination resources, but this response is not the optimal resource allocation for subsequent fruit development and seed maturity, which suggests that the response of plants to external environment resources is short-term and direct.

## 1. Introduction

Responses by the plant’s reproductive system to external resources and the environment are central to the study of plant evolution [1,2]. Plant fitness is an important means of evaluating plant responses to environmental factors [3,4]. Plant fitness serves as an index for evaluating the adaptability of plant characteristics in specific environments and the contribution of individual plants to producing the next generation [5]. As the resources needed for the growth of roots and leaves, flowering, fruiting, and seed production are not the same, the plant’s investment in these activities is not fixed [6,7], and also, the resources are finite and result in life history trade-offs in plants [8]. To achieve optimal fitness, plants have many trade-off strategies in resource acquisition and distribution. Floral display is fundamental to plant fitness; it will affect pollinator visitation rate and total seed production [9]. In plants, the pool allocated to floral function is limited; there should have some trade-offs among behaviors in floral display [10,11]. For example, there are trade-offs in reproductive allocation between flower size and number [12], also fruit weight and seed number for the optimal fitness [13,14].

For monoecious plants, the trade-offs to a certain limited resource should also include the differentiation of male and female flowers [15,16], because the resources needed for the growth of male and female flowers are different, and the production of male flowers requires less investment than the production of female flowers [17,18]. When the environment has abundant pollen resources, the fitness of female functions is greater than that of male functions [1]; therefore, the plant will increase investment in female functions, such as increasing the number of female flowers, increasing the nectar yield and extending the flowering period [19,20]. When the nutrient resources are sufficient, more ovaries may develop into fruit, and when the nutrients are limited, the development of more ovaries may be blocked, and even fruit drop rate increases [21]. Furthermore, this will affect the allocation of male and female flowers in plants. When female flowers are pollinated in order to increase their reproductive fitness, the plant will correspondingly supplement the number of female flowers and decrease the proportion of male flowers [22]. Bateman’s principle states that male fitness is usually limited by the number of mating achieved, while female fitness is usually limited by the resources available for reproduction [23]. Pollen availability may limit female success with respect to individual flowers, entire plants (in a season or over a lifetime), or populations [19,20]. However, the shortage of nutrient resources can make a plant increase its investment in male functions because that investment may have bigger returns than in female functions [1]. The other side of increasing investment in female functions is that the production of fruit then requires more nutrient resources. For the development of seeds, more seeds mean more resources are required. However, too much fruit and seed production can lead to a reduction in nutrients allocated to each fruit [24,25], which goes against the optimal resource allocation of heredity, i.e., the fitness of offspring is limited [26,27]. This status may be more significant when environmental resources are restricted [28]. Therefore, there is a trade-off between the production of female flowers and the production of single fruits or seeds. Male flowers require far fewer resources than female flowers, so in the low nutrient conditions or fewer pollen resources, male flowers may increase the fitness of plant reproductive success more than female flowers to maximize the allocation to resources. Consequently, from a plant perspective, the production of cheap male flowers is an economic and effective strategy [18,29]. However, plants cannot regulate the ratio of male and female flowers from the perspective of the whole life cycle. When resources for pollination are not limited, it is not clear whether a plant will produce a limited number of female flowers to bear larger fruits that may yield better seeds or whether more female flowers will be produced to maximize the use of resources.

Cucumber (*Cucumis sativus*: Cucurbitaceae) plants are known for their plasticity in sex expression and are ideal for studying the effects of pollinators on plant performance [30]. Cucumber is a kind of monoecious plant, and also the sexual expression of cucumber is susceptible to the influence of environmental factors such as light, temperature, or hormone levels [29,31,32,33,34,35]. The sexual expression of cucumbers is highly sensitive to the surrounding environment including internal and external factors. Cucumbers can adjust their sexual expression to achieve the maximum ability to use and allocate environmental resources [31]. Total flower production varies according to the growing conditions, but the number of male flowers are usually 3–4 times that of females [36]. However, it was not clear whether the pattern of male and female flowers in the flowering period of cucumber is still beneficial to fruit production and seed ripening in the future. An attempt was made in this research to study the response of the sexual expression of cucumbers to the availability of pollen under controlled soil–nutrient conditions, specifically, (1) the impact of artificial pollination for improved fruit and seed set in cucumbers and (2) whether there is a trade-off between female flower production and fruit or seed production in response to nutrient allocation and pollen resources.

## 2. Results

### 2.1. Total Number of Male and Female Flowers

**Figure 1:**
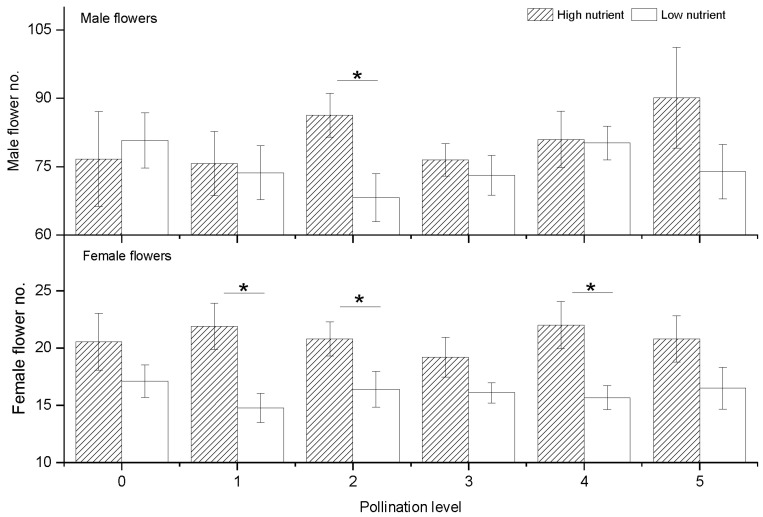
Total male and female flowers for 32 days per plant under different nutrient and pollination treatments. Plots show mean ± SE, *n* = 10, and asterisks (*) above the columns show significance between high and low nutrient treatments analyzed using one-way ANOVA.

The results showed that the total number of male and female flowers in cucumber was significantly higher under high nutrient conditions than that in low nutrient conditions, but pollination intensity did not significantly increase the total number of female and male flowers (Table 1). Further, it was found that nutrients had less effect on male flowers than female flowers, but after pollination stopped, the high nutrient level could significantly increase the number of male flowers (on the 43rd day) (Table 1). This suggests that after the pollination experiment, the plant significantly increased male flowers. Statistics showed that although pollination has little effect on the total number of male flowers, it can significantly affect the number of male flowers of cucumber per day. Specifically, we found that when pollination P2 level was given, high nutrients would significantly increase the number of male flowers of cucumber (Figure 1). Unlike male flower, the response of female flowers to nutrients was more obvious, the number of female flowers under high nutrient conditions was higher than that under low nutrient conditions (Table 1), and it was statistically significant under the treatment of pollination levels P1, P2, and P4 (Figure 1), indicating that the highest number of female flowers did not correspond with the highest pollination level (Figure 1).

In addition, we also analyzed the differences in the male/female ratios of flower number, finding that the male/female flower ratio was slightly higher in low nutrient levels than in high nutrient levels (the ratio was 2.25 ± 0.0059 in low nutrient, and in high nutrient the ratio was 2.23 ± 0.0058), but the pollination treatment can significantly affect the ratio of male: female flowers per day (Table 1), indicating that cucumber will gradually increase the proportion of male flowers in the flowering stage.

### 2.2. Dynamic Changes in the Number of Male and Female Flowers

**Figure 2:**
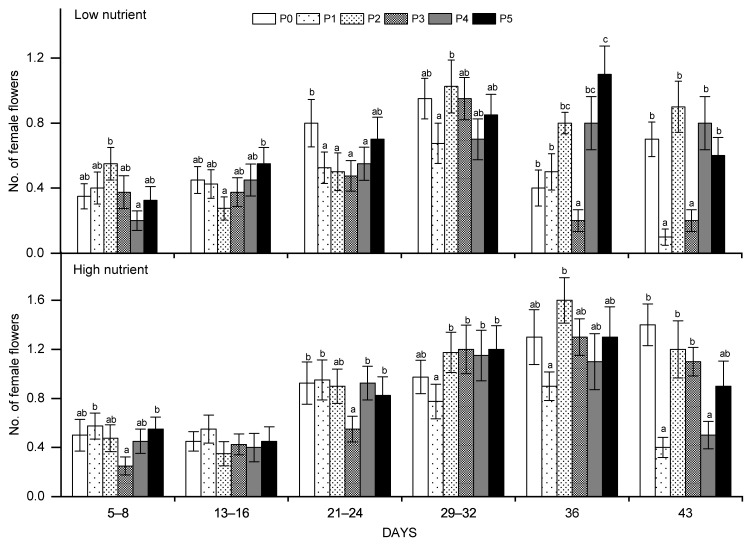
The average number of female flowers counted every 4 days with an interval of 4 days during the pollination treatments (in 32 days) and the 2 records of female flowers after the pollination treatments stopped (36 day and 43 day). Plots show the mean ± SE, *n* = 10, and the different letters (a, ab, b) above the columns indicate significant differences among different pollination levels. No letters indicate that the number of female flowers at this pollination level was not different from that of other pollination levels. The data were divided into high and low nutrient groups and analyzed with one-way ANOVA and the Tukey test for multiple comparison testing.

Further, the number of male flowers per day was positively affected by pollination, and the number of new female flowers increased significantly every day affected by nutrient or pollination, or their interactions (Table 1). Obviously, with the growth of cucumber plants, the number of female flowers increased gradually during pollination days (repeated measures ANOVA, df = 31, F = 15.008, *p* = 0.000 < 0.05) (Figure 2). Moreover, the number of female flowers reached the highest on the 4th day after pollination experiment stopped (i.e., the 36th day of the pollination experiment), while the number of female flowers showed a significant decrease on the 11th day (43 days) compared with the 4th day (36 days) in the low and high nutrient levels (*t*-test, df = 19, t = 2.269, *p* = 0.035 < 0.05), but the number of flowers in the control group without pollination did not decrease significantly. By comparing the changes in the number of female flowers between pollinated and non-pollinated, we can find that the number of female flowers showed a significant decline after pollination stopped, and also there was a significant difference between different pollination levels (Figure 2). Furthermore, from the difference in the number of female flowers two times after the pollination experiment was stopped, it shows that pollination may have a persistent effect on the increase in female flowers in cucumber.

If we consider the effect of daily pollination on the number of female flowers, we found that there were significant effects in low or high nutrient levels, which mainly appeared during the later stages of the pollination experiment (Figure 2). In addition, different pollination levels lead to differences in the peak number of female flowers in low or high nutrient levels during the pollination experiment (i.e., in high nutrient level) with the increase in the pollination degree, the number of female flowers gradually increased in the late stage of the pollination experiment (Figure 2).

### 2.3. Reproduction Set

**Figure 3:**
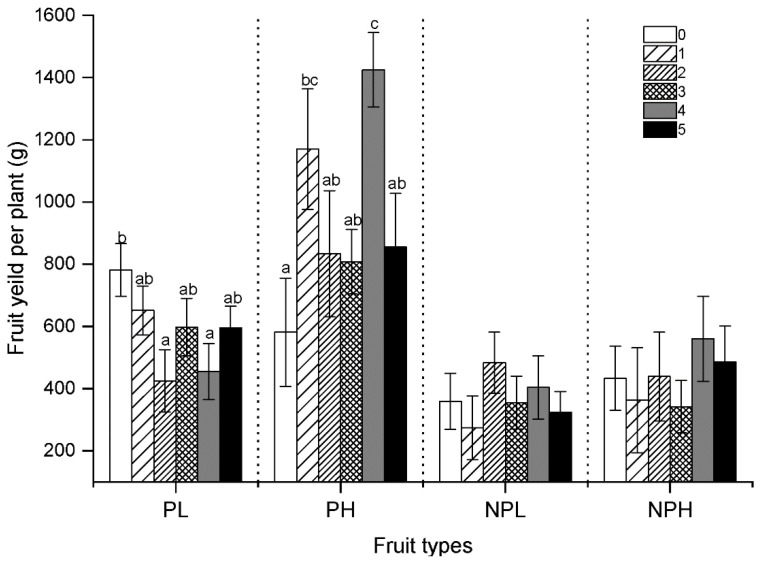
The fruit yield (pollinated and non-pollinated) of cucumber grown under two nutrient conditions changed with pollination gradient. PL (pollinated under low nutrient), PH (pollinated under high nutrient), NPL (non-pollinated under low nutrient), and NPH (non-pollinated under high nutrient). Plots show mean ± SE, *n* = 10, and different letters (a, b, c, ab, bc) indicate significant differences, analyzed using one-way ANOVA with the Tukey test for multiple comparison testing.

**Figure 4:**
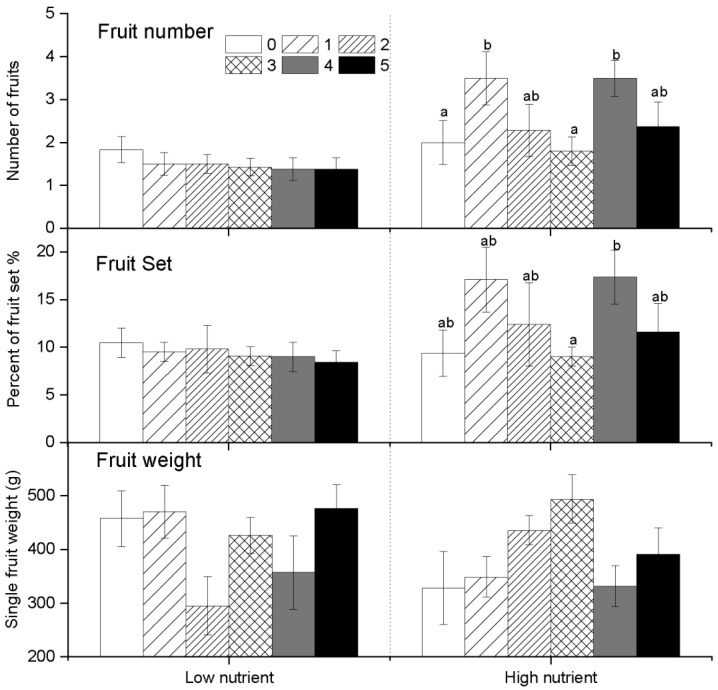
Under two nutrient conditions, the fruit production (fruit number, fruit set percentage, and fruit weight) changed with pollination levels. Plots show the mean ± SE and different letters (a, ab, b) indicate significant differences among different pollination levels analyzed using one-way ANOVA with the Tukey test for multiple comparison testing.

Cucumbers can also bear fruits without pollination; however, our results showed that nutrient and pollination treatments significantly affected the fruit yield during the pollination experimental stage. After the pollination experiment, although artificial pollination was stopped, these plants still produced fruits, but there was no significant difference in fruit yield among plants (Table 2, Figure 3). Our results showed that under low nutrient conditions, the highest fruit yield occurred at the level of no pollination (P0), but under high nutrient conditions, fruit yield increased with the pollination level. Therefore, pollination can increase fruit yield only when nutrients are sufficient and may reduce fruit yield when nutrients are insufficient (Figure 3). Similarly, under low nutrient conditions, pollination did not increase the number of fruits and fruit set percentage per plant. Even from the average value, pollination reduced the number of fruits and fruit set percentage (Figure 4). However, under high nutrient conditions, pollination can promote plants to produce more fruits and have a higher fruit set percentage (Figure 4). Although high nutrient can increase the fruit set of cucumber, combined with the number of female flowers (Figure 1), we can see that the percentage of fruit set was still low, only around 10%. This also indicated that a large number of female flowers fail to produce fruit. Pollination leads to an increase in female cucumber flowers, but the plant does not guarantee that all pollinated female flowers develop into fruit, thus resulting in a large number of fruit abortion. Therefore, the effect of pollination on the weight of a single fruit was different from that of fruit number and fruit set percentage. Compared with fruit number and fruit set percentage (such as at P3), the weight of fruit was higher at P3 than others (Figure 4). This showed that high pollination may lead to higher fruit number but reduce the weight of a single fruit. Similarly, nutrients increased fruit number and fruit set percentage, but did not improve the fruit weight. The patterns of fruit number and fruit set were similar to those of the yield of fruits; the values were greatest at the P1 level, and there was a small downward fluctuation with the increase in pollination levels (Figure 4). This means that a continued increase in pollination will lead to excessive pollination for a plant, and then may reduce the number of fruit, thus decreasing the yield of fruits per plant. So that, because the fruit number decreases, the average weight of a single fruit will increase (P3) (Figure 4).

**Figure 5:**
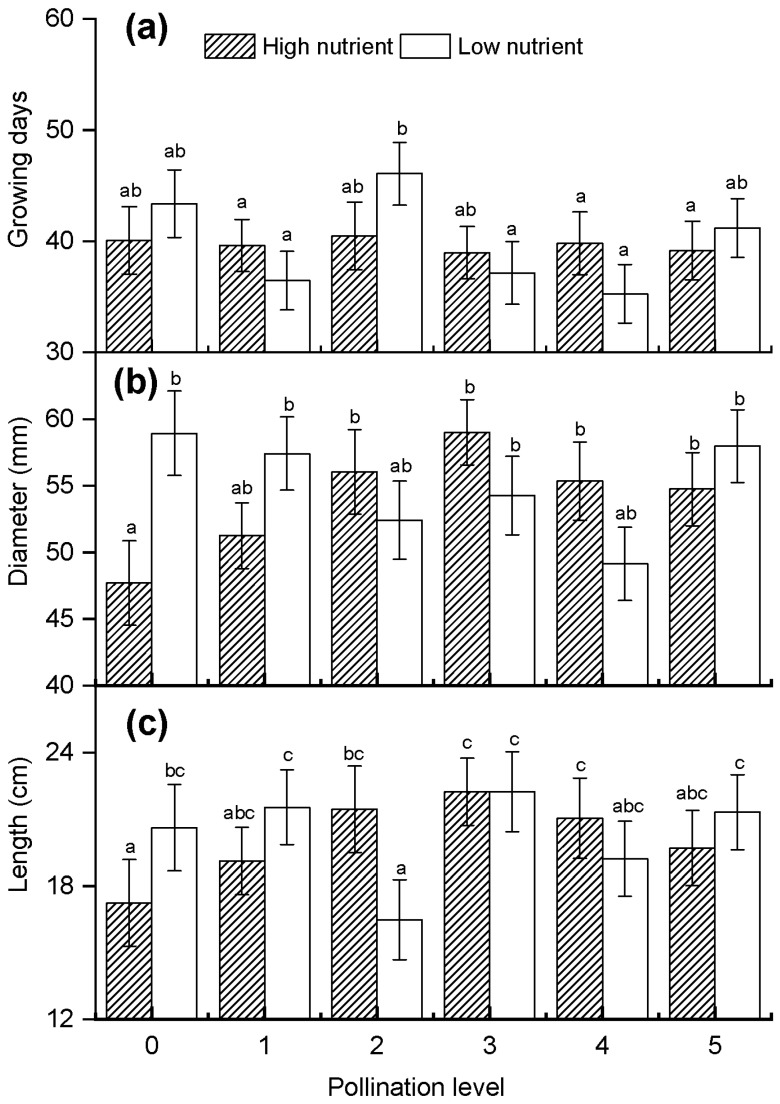
(**a**) Number of days required for fruit to reach maturity and (**b**) fruit diameter and (**c**) fruit length per fruit under different nutrient and pollination treatments. Plots show mean ± SE and different letters (a, b, c, ab, bc, abc) indicate significant differences analyzed using one-way ANOVA and the Tukey test for multiple comparison testing.

The days of growing and the fruit size (diameter and length) were also recorded. The results indicated that the effects of the nutrient and pollination treatments were not significant on the days of fruit growing from pollinated to harvest (Table 2, Figure 5a), but at high nutrient level, the growing days appeared to be more stable than that at low nutrient level (Figure 5a). Though from the whole effects of nutrient and pollination, there were no statistical differences on fruit diameter and length (Table 2), we found that under high nutrient condition, the data of diameter and length of fruit have a peak, both in P3 pollination level reached maximum, and the minimum in the control of pollination (P0), in the levels of pollination P4 and P5, fruit diameter and length were gradually decline (Figure 5b,c). This suggests that high intensity pollination did not increase the size of single fruit. In addition, at low nutrient levels, the effect of pollination on fruit diameter and fruit length was slightly opposite to that at high nutrient levels (Figure 5b,c). There was a trough at the level of pollination P4 in fruit diameter and P2 in fruit length. Thus, this suggests that pollination (P3) made the fruit thinner and longer in nutrient abundance, while in nutrient deficiency, pollination made the fruit thicker (in P4) and shorter (in P2) (Figure 5b,c).

### 2.4. Seed Production

**Figure 6:**
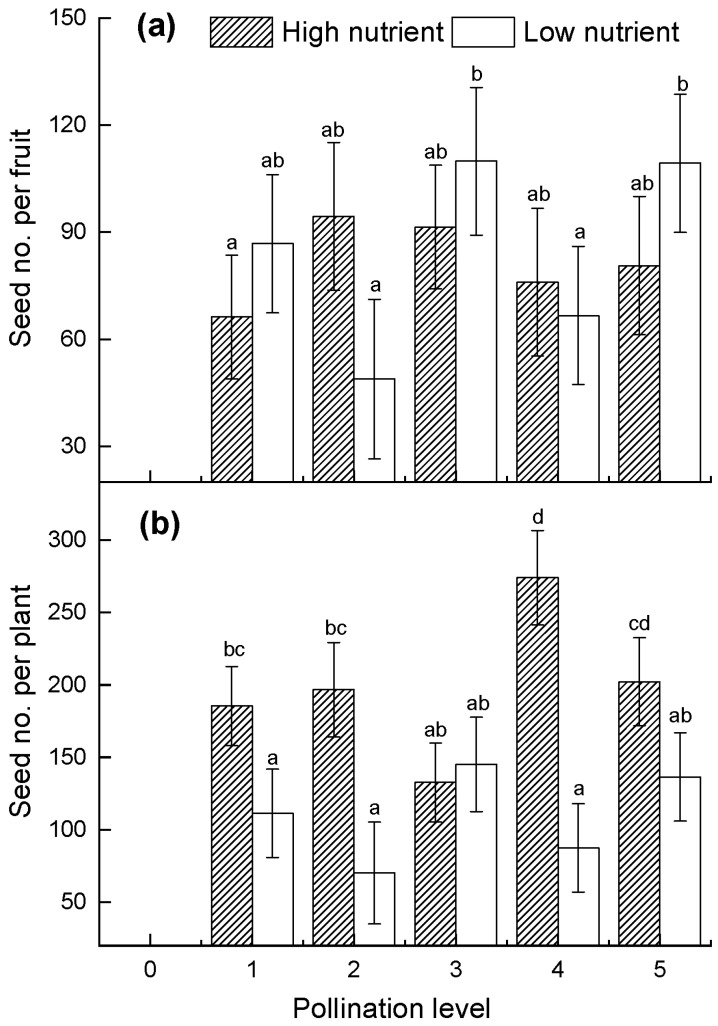
(**a**) Number of seeds per fruit and (**b**) total number of seeds per plant under different nutrient and pollination treatments. No seeds were produced in the non-pollination experiments (P0), so the plots at P0 were empty. Plots show mean ± SE and the different letters (a, b, d, ab, bc, cd) indicate significant differences analyzed using one-way ANOVA and the Tukey test for multiple comparison testing.

We harvested all the fruits and counted the number of seeds for each fruit. The results showed that the seed production was significantly affected by nutrients and pollination. Obviously, no seeds were produced in the non-pollination experiments; thus, the plots were empty (Figure 6a,b). At high nutrient levels, the number of seeds per fruit was higher at the P2 and P3 levels than in the control (Figure 6a). At the low nutrient level, however, the number of seeds per fruit at the P2 level was almost the lowest. Unlike seed number per fruit, the total number of seeds per plant was significantly affected by the interaction between nutrients and pollination (Table 1) and differed from the number of seeds per fruit among pollination levels under low nutrient treatment (Figure 6b). By contrast, the lowest value was observed at the P2 level, but an increase was observed at the P4 level (Figure 6b).

### 2.5. Correlation between Flowers and Fruits and Seeds

Correlation analysis showed that the female flowers were positively correlated with the number of male flowers, number of fruit, fruit yield per plant, total number of seeds per plant, and the shoot mass but were not correlated with the weight of fruit and the number of seeds per fruit (Table 3). Moreover, the number of fruit per plant was positively correlated with fruit yield and the total number of seeds per plant but was uncorrelated or negatively correlated with the weight of single fruit, the number of seeds per fruit, and the shoot mass. Additionally, the fruit yield per plant was also related to the total seed number per plant (Table 3). These results showed that the number of fruits increased with the number of female flowers and that the fruits per plant had a greater yield and a higher number of total seeds; however, there was also a decline in the weight of fruit (Figure 4) and the number of seeds per fruit. Accordingly, the total number of seeds per plant was negatively correlated with the number of seeds per fruit, and the number of seeds per fruit was positively correlated with the fruit weight (Table 3). These results showed that a high number of fruit per plant can lead to reductions in the weight of a single fruit and that smaller fruit produces fewer seeds. Additionally, the shoot mass was negatively correlated with the number of fruit and fruit yield, and the total number of seeds showed a trade-off between vegetative growth and reproductive allocation (Table 3).

## 3. Discussion

The responses of gender expression of monoecious plants to different environments are plastic [37,38]. In a nutrient-rich environment, plants tend to increase investment in sexual reproduction [39,40]. In this experiment, cucumbers produced more female flowers at high nutrient levels. Therefore, under the condition of sufficient nutrients, cucumber chooses to increase the number of female flowers to maximize resource income. In addition to nutrients, pollen and pollinators should also be regarded as an external resource; the richness of these resources can affect plant flowering behavior [41]. Studies have shown that increases in pollinators, such as bees, can increase the number of fruits per plant, thereby increasing the crop yield as well [42,43]. In this study, the number of female flowers changed to a certain degree with the level of pollination. The study also illustrated the influence of the strength of pollination on the differentiation of female flowers. The peak number of female flowers did not appear at the highest pollination level but at middle levels, which suggests that the response of cucumber female flowers to the intensity of pollination is not a linear increase but will increase or decrease based on the nutrient supply. From the flowering period until the end of the pollination experiment, the number of female flowers gradually increased; this was associated with the growth of the plants, but the pollination experiment showed that in the late stage of pollination, the number of female flowers differed significantly among different pollination levels, which suggested that the effect of pollination on plant flowering behavior was mainly on the number of new flowers. This further illustrated that pollination treatment had an accumulated effect on cucumber flowering behavior. Increasing the pollination makes plant flowers subsequent increase in number, but when pollination stopped, cucumber will reduce the flowering (Figure 2); it also illustrated that the response of flowering behavior of cucumber to pollen resources is subsequent and short-lived. However, studies on the flowering behavior of plants are less about the response to pollination timeliness and more about the responses to the spatial and temporal distribution pattern of pollen resources [44,45].

The production of male flowers is easier and cheaper than that of female flowers; therefore, plants will produce more male flowers for maximum fitness when resources are sparse [17,46]. There were also studies show that the resources saved by producing male flowers are not re-allocated to other fitness enhancing functions [18]. In this study, there were significantly fewer female flowers under low-nutrient conditions, but the number of male flowers remained unchanged. In this way, the ratio of male/female flowers increased, proving that male fitness increases under resource-poor conditions [47]. Furtherly, during the pollination experiment, the ratio of male/female flowers of cucumber increased significantly with days, which also indicates that the annual plants in the late stage of flowering will increase the ratio of male flowers for increasing their nutrient returns and improving their fitness.

In addition, under the condition of low nutrient, low concentration of pollination can stimulate the change of female flower number. After the experiment of pollination, cucumber significantly reduces the number of female flowers, which also indicates that cucumber will reduce the investment in female flower function under the condition of limited pollen resources (Figure 2); however, the pollination treatment did not affect the number of male flowers, which did not decrease at different pollination levels, even after the pollination experiment, the number of male flowers didn’t decrease significantly. Thus, to the fitness of plant, it is a kind of complementary effect on the number of female flowers that decreased under low nutrient conditions after pollination. This compensation effect enables plants to make optimal use of resources such as nutrients and pollen, and to maintain the maximum fitness of plants [48,49].

As we know, there are differences between fruit production and seed production, because the former comes is attributed to the ovary of a cucumber plant, and seed production requires the ovule to develop normally after fertilized. For this reason, fruit growth in some cases is not restricted by pollination; rather, it may be more conducive to the production of fruit if no pollination occurs [50]. In this experiment, the pollination treatment significantly increased fruit yield per plant (Table 2), which was consistent with the number of female flowers and peaked at the P1 level. In our experiment, the relative number of setting fruit exhibited a trend similar to that observed for the number and yield of fruits, which shows that a high ratio of fruit set may be associated with a high number of fruit as well as a high fruit yield. However, these trends did not increase with the levels of pollination but peaked at the P1 level and then fluctuated with a declining trend, which suggests that excessive pollination can lead to restrained fruit growth and can thereby decrease the relative amount of setting fruit. The percentage of fruit set was the lowest in the control pollination group, but some fruits did develop, which proved that the growth of cucumber fruit is not entirely affected by pollination.

A high pollination level may thus increase the total number of cucumber seeds per plant, but this can lead to a decrease in the number of seeds per fruit. However, this trend changed when the level of soil nutrients was low; plants increased their investment in seed development, although the total number of seeds was not higher than at high nutrient levels, and the number of seeds per fruit was significantly higher at the high pollination levels, i.e., P3 and P5. That is to say, under the condition of sufficient nutrients, increasing pollination will increase the total seed number per plant, but very high levels of pollen will lead to a reduction in the seed number per fruit, while under the condition of insufficient nutrients, pollination will increase the seed number per fruit. Cucumbers thus tend to allocate more nutrients to sexual reproduction to produce more seeds when resources from the external environment are sparse. When resources are abundant, the proportion of investment in the seed set decreases, although the total number of seeds per plant increases because of the increasing number of fruit. During the flowering stage, cucumbers coordinated the ratio of male and female flower number based on the soil nutrient conditions and the intensity of pollination. This trade-off allows cucumbers to maximize their use of resources. Here, from the perspective of the differentiation of male and female flowers, cucumbers maximized the reproductive fitness. However, the growth of fruit and seed development require nutrients, and the demand for nutrients differs during the flowering stage because the production of male and female flowers is also determined by nutrients and pollination. The pattern of male and female flowers in the flowering stage, which optimizes the use of nutrients and pollens, may not realize the maximum fitness during the fruiting or seed setting stages. The current study suggests that the number of female flowers is positively correlated with the number of fruits and the total number of seeds but not the weight or number of seeds per fruit. In this way, more female flowers mean more fruits, which can lead to smaller and less developed fruits. The trade-off of plants in the flowering stage may not favor fruit maturity or seed development.

In our study, the effects of pollination and nutrient treatment on cucumber should be different. Pollination levels will directly affect seed development, and soil nutrient levels will directly affect the number of male and female flowers. However, these two types of patterns are different in their demands for resources, and there is a trade-off between seed and flower production. For this reason, the number of female flowers was not necessarily positively correlated with the number of seeds per fruit in this study. The number of flowers was established during the blooming phase based on the conditions of soil nutrients and other possible factors; however, the number of male to female flowers may not be the optimal arrangement for fruit growth and seed maturation, and the pattern of more flowers, will not necessarily lead to larger fruits and more seeds. In this experiment, the number of female flowers was consistent with the number of fruit and the total number of seeds but not with the fruit yield and the number of seeds per fruit, which means that more female flowers will have more fruits, although smaller fruit with fewer seeds. In Cucurbitaceae crops, cultivators may remove secondary-lateral branches to prevent the production of too many female flowers [51]. This may be particularly relevant to watermelon management, where this practice may ensure bigger fruit, especially considering that people usually keep only one fruit per plant [52,53]. An increased production of female flowers may later require more resources for increased fruit and seed production. This may not benefit the development of good seeds because fewer resources are allocated to a single fruit. Fitness of plants may be reduced in this manner. Of course, cucumber is a kind of vegetable. No seeds or few seeds are considered to have better economic value and quality, but only for cucumber itself, its maximum reproductive fitness should be to maximize the investment in the next generation and producing more seeds should be an important consideration index of its fitness. For farmers, in cucumber planting, reducing pollination to obtain seedless fruit may be our pursuit. However, if we want to get more mature cucumber seeds, increasing pollination may maximize the benefits.

Plants must respond to the external environment by allocating their resources for specific goals. Evaluating the fitness of this type of immediate response does not get high scores. For example, large numbers of seeds often germinate from a single fecal clump, although competition may leave many delicate seedlings [54]. The response of plants to the external environment or resources is usually short-term. For example, if fertilizer is added, plants will grow rapidly, and if light is increased, plant photosynthetic products will increase quickly. Plants will not be reasonably allocated according to these external environmental conditions or resources from the perspective of the whole plant life history. So, it may be disadvantageous to the growth of the next generation to evaluate the fitness or adaptation to the environment [54]. However, in the long-term evolutionary process, plants have some strategies that may make up for defects. For example, plants can adjust the number of fruit after flowering by dropping fruit to adapt to the nutrient availability [55]. In this study, cucumbers also dropped many fruits after flowering. From this perspective, dropping of fruit may be a type of regulation of fruit growth in response to environmental conditions. Dropping of fruit also illustrates that the layout of plants with respect to the number of flowers during the flowering stage may not be suitable for the growth of fruits and seeds during a later stage.

## 4. Materials and Methods

### 4.1. Materials

Cucumber (*Cucumis sativus*: Cucurbitaceae) is widely cultivated annual plant. The male flowers, which are solitary, grow from leaf axils. Female flowers of cucumber are similar to the male flowers but have a spindle-shaped ovary underneath. In general, cucumber is monoecious and reliant on pollinators for fertilization. Both male and female flowers remain open for a single day and are visited by a variety of generalist pollinators [30]. The cucumber fruit is cylindrical and approximately 10–30 cm long. Cucumbers require well-drained soil with 80–90% humidity for growth, and they require 60–70% substrate humidity after the seedling stage.

### 4.2. Cultivation

Wild cucumbers have various phenotypes, and they show different shapes even under the same conditions of cultivation. Therefore, in order to reduce experimental errors, artificial domesticated varieties were adopted in this study. All cultivation and experiments were conducted in the College of Natural Sciences greenhouse (CNS greenhouse) of Massachusetts, USA. At first, 120 cucumber seeds (*Cucumis sativus*; Marketmore 76, Johnny’s Selected Seeds Company, Fairfield, ME, U.S.) were sown in the cell trays in the greenhouse at maximum and minimum temperatures of 24 and 18 °C, respectively, using ProMix HP potting soil (Pro-Mix HP Mycorrhizae Company, Québec, QC, Canada). All the plants for experiment were placed randomly in greenhouse cultivation platform, and they can accept sunlight severally. To avoid the error caused by location, we made all the labels of serial number according to the levels and replications of nutrient and pollination treatment, and upset the sequence of serial number, then randomly pasted on each pot of cucumber. We treated fertilizer and pollination according to the label on the pot. Plants were watered daily as needed. After the seedlings grown at least two true leaves, each seedling was then transplanted into a 4” pot (0.57 L), also using ProMix HP potting soil. On 22 December of the same year, Marathon II (OHP Company, Vineland, NJ, USA) was used as a soil drench at a rate of 1.6 mL/L to control insect pests.

### 4.3. Experimental Design

The experiment consisting of fertilizer (2 levels) and pollination (6 levels) concentrations was carried as a full factorial design. There were 10 replications for each of the 12 treatment combinations, resulting in a total of 120 plants. Fast-release Osmocote fertilizer with a 20:20:20 N:P:K ratio (The Scotts Company LLC, Marysville, OH, USA.), was initially added at concentrations of 150 and 300 ppm, respectively, when the plants were still in the seedling stage, i.e., before final transplantation [56,57]. When the plants had 5–6 leaves, each seedling was transplanted into a 7.57 L pot, and fast-release fertilizer was added once based on the previous nutrient settings. After one week, Osmocote slow-release fertilizer was added (N:P:K ratio 14:14:14) at a rate of 12 g per plant in the high-nutrient treatment group and 6 g per plant in the low-nutrient treatment group. These levels were consistent with Osmocote fertilizer guidelines for potted plants.

Pollination treatments commenced shortly after the female flowers appeared. An additional 20 non-experimental cucumber plants were also planted to serve as pollen donors. Each pollen donor was grown and fertilized in the same way as plants in the high-nutrient treatment group.

### 4.4. Male and Female Flower Production

The numbers of male and female flowers were recorded daily once plants began flowering. Because cucumber flowers open in the morning and wither the following morning, daily counts were strongly indicative of male and female flower production. All flowers were counted until the end of the pollination treatment (32 days). Additionally, the number of flowers was recorded twice to assess the persistent responses of cucumbers, and the records was at the 4th day and the 11th day after the end of the pollination treatments, i.e., that is the 36th and 43rd day from the start of the pollination experiment.

### 4.5. Pollination Settings

Previous studies on cucumber pollination generally placed the pollen directly on the flower with a toothpick or a cotton paintbrush [58]. However, this method is not conducive to accurate control of the pollen concentration. Instead, we used a sucrose-pollen solution that has been used in other plants [59,60]. The anthers were collected from 10 male flowers from the pollen donors each day. All stamens were cut and placed in a tube with 10 mL of 10% sucrose solution [60], and the tube was then vortexed for 2 min. This solution was used for the highest level of pollen concentration (Level 5) and was serially diluted to create the 4 lower pollen concentration solutions. For each dilution, the volume of solution was doubled (and the concentration of pollen was thus halved) by adding an equivalent amount of sucrose solution to a portion of the pollen solution. A hemocytometer was used to quantify the concentration of pollen in 5 samples from the highest concentration solution [61,62]. The average number of pollen grains in one square (1 mm^2^) was 2.2 ± 0.84 grains, and the concentration from the formula was 110 grains/μL. All female flowers on each plant were pollinated every day for one month by placing 20 μL of the pollen solution on the stigma by pipette. In the highest pollination treatment, the total number of pollen grains was 2200 (P5); accordingly, the total number of pollen grains was expected to be 1100 (P4), 550 (P3), 275(P2), and 137.5 (P1) grains for other treatment levels and zero in the controls (20 μL sucrose solution without pollen) (P0). Each pollinated female flower was labeled with the date of pollination to allow calculation of the days required for fruit development. The pollination treatment was performed from 8:00a.m. to 10:00a.m. every day for 32 days during flowering stage.

### 4.6. Fruit Production

All fruits were harvested when they reached a length of 18 cm or one month after the end of the pollination treatments, whichever indicated maturity, beyond this length, or after one month when fruits began to turn yellow and decay [52]. All the fruits were recorded after the pollination experiment. Some female flowers produce fruits even when they are not pollinated, so we also harvested the fruits of the control group that are not pollinated. At harvest, the date, fresh mass of fruits (fruit yield) per plant, fresh weight of per fruit, length, and diameter of each fruit were recorded. Then, the fruits were cut in half length-wise, the seeds visible in both halves were counted, and this value was averaged, first per fruit and then per plant [30]. The seed counts were used to evaluate the ability of the fruit to produce seeds. Fruit set was calculated as the proportion of female flowers that produced fruit. Fruit yield, fruit length, diameter, and seed counts were averaged per plant so that each plant could be treated as a unit of replication. In addition, in order to further explore whether plants exhibit plasticity regulation between reproductive growth and vegetative growth in response to nutrients and pollination treatment, we harvested the aboveground portion of plant. After fruit harvest, all the above-ground parts of the plants were harvested, air-dried in sunlight for 15 days, and weighed as shoot mass.

### 4.7. Data Analysis

Results were analyzed using general linear models (GLM, SAS 9.3; SAS Institute, Cary, NC, USA) for numbers of male and female flowers, fruit yield per plant, fruit weight, fruit size, number of seeds, and above-ground plant mass, and nutrient and pollination treatments and their interactions served as independent variables. Post hoc comparisons between treatments were conducted with Tukey’s HSD test. Response variables were averaged per plant so that individual plants could be treated as units of replication. Three plants that were sick and grew poorly during the experiment were excluded. All the response variables were transformed to meet the assumptions of normality and homogeneity of variance by square root transformation, seed number was square root transformed, and fruit number was arcsine square root transformed. Normality was confirmed and homogeneity of variance verified with Levene’s test. We took the un-pollinated cucumber as the control and carried out variance analysis on the difference between the number of female flowers of cucumber under each pollination level and the control, and we tried to analyze the difference in the change number of female flowers of cucumber under different pollination levels. Here, we used one-way repeated measure ANOVA to analyze the differences of the changes of female flowers in each nutrient level, with only pollination as independent variables, and the number of daily changes in female flowers as dependent variables, the factor of days is repeated measure factor. In addition, just to make it a little bit more intuitive, we plotted the average number of flowering every four days of pollination treatment and observed the change in the number of female flowers with days. Correlation analysis of cucumber flower, fruit, seed traits, and shoot mass was based on Pearson’s correlation coefficients. The results are presented as non-transformed means. The significance values in the text are at *p* < 0.05, unless stated otherwise.

## 5. Conclusions

In this paper, we would like to discuss the response of plant reproduction and nutrient distribution strategies to the external environment (nutrients and pollination). According to our traditional view, plants always achieve the optimal allocation of resources. However, this kind of distributive response of the plant is not considered from the whole life history of the plant, and it is often an immediate response to the external environment, at least to nutrient and pollination in the experiment. We see in the results that the number of female flowers could increase along with the increase in nutrient concentration, and it also achieved a higher level under a certain degree of pollination, but this pattern of resource allocation of male and female flower numbers to nutrients and pollination resources was not the most suitable for subsequent fruit growth and seed development of. Therefore, this study suggests that plant response to the external environment is not a long-term response from the perspective of overall resource allocation. Response to the external environment can be described as an instantaneous stress reaction. This stress response is also meaningful from an evolutionary perspective, and this plasticity of regulation allows organisms to adapt to changes in their environment [55,63,64].

However, our conclusion is based only on the study of domesticated cucumber varieties. We still do not know whether wild plants or wild cucumbers under natural conditions will be more adaptable to environmental resources than artificial varieties. This information is needed to properly adjust the allocation of resources between flowering and fruit development. The flowering and fruit production of wild plants are more sensitive to the external environment than domesticated varieties because of their higher number of phenotypes [65], which may further verify the immediacy of plant response to environmental resources. However, more research on wild plant species is needed to confirm this.

## Figures and Tables

**Table 1:** ANOVA showing the differences in numbers of male flower female flower, ratio of male/female flowers among different nutrient and pollination treatments. Pollination treatment lasted for 32 days, and the 36th and 43rd day were the records after pollination treatment.

Factors	Dependent v	Df *	F *	*p*-Value
Nutrient	Total number of male flowers	1	4.80	0.031
	Total number of female flowers	1	21.47	0.000
	Number of male flowers per day	32	1.48	0.084
	Number of female flowers per day	32	1.68	0.033
	Number of male flowers on the 36th day	1	2.50	0.117
	Number of male flowers on the 43rd day	1	10.60	0.002
	Number of female flowers on the 36th day	1	10.52	0.002
	Number of female flowers on the 43rd day	1	5.05	0.027
	Ratio of male/female flowers per day	32	0.943	0.562
	Ratio of male/female flowers on the 36th day	1	1.01	0.317
	Ratio of male/female flowers on the 43rd day	1	0.35	0.554
Pollination	Total number of male flowers	5	0.57	0.72
	Total number of female flowers	5	0.15	0.98
	Number of male flowers per day	32	2.30	0.001
	Number of female flowers per day	32	1.61	0.045
	Number of male flowers on the 36th day	32	0.97	0.437
	Number of male flowers on the 43rd day	5	1.82	0.115
	Number of female flowers on the 36th day	5	0.88	0.501
	Number of female flowers on the 43rd day	5	2.25	0.037
	Ratio of male/female flowers per day	32	1.60	0.047 *
	Ratio of male/female flowers on the 36th day	5	0.59	0.708
	Ratio of male/female flowers on the 43rd day	5	0.29	0.919
Nutrients and Pollination	Total number of male flowers	5	0.80	0.554
	Total number of female flowers	5	0.15	0.98
	Number of male flowers per day	32	1.42	0.104
	Number of female flowers per day	32	2.15	0.003
	Number of male flowers on the 36th day	5	0.20	0.962
	Number of male flowers on the 43rd day	5	1.27	0.284
	Number of female flowers on the 36th day	5	0.62	0.688
	Number of female flowers on the 43rd day	5	1.07	0.382
	Ratio of male/female flowers per day	32	2.38	0.001
	Ratio of male/female flowers on the 36th day	5	0.44	0.819
	Ratio of male/female flowers on the 43rd day	5	1.06	0.384

* df means Degree Freedom. F is the statistical value of ANOVA.

**Table 2:** Two-way ANOVA showing the differences in fruit yield per plant, number of fruits, fruit set, fruit size, fruit weight, and number of seeds among different nutrient and pollination treatments. The data on fruit yield per plant (non-pollinated fruits) are statistics of the fruits produced after stopped the pollination experiment, and the data on fruit yield per plant (pollinated fruits) are statistics of the fruits produced during pollination experiment.

Factors	Dependent Variables	Df *	F *	*p*-Value
Nutrient	Fruit yield per plant (pollinated fruits)	1	22.7	0.000
	Fruit yield per plant (non-pollinated fruits)	1	1.20	0.278
	Weight of fruit	1	0.06	0.810
	Number of fruit	1	18.11	0.000
	Percentage fruit set	1	7.03	0.009
	Days of fruit growing	1	0.15	0.702
	Diameter of fruit	1	0.23	0.635
	Length of fruit	1	0.04	0.839
	Total no. seeds per plant	1	15.06	0.000
	Number of seeds per fruit	1	0.09	0.766
Pollination	Fruit yield per plant (Pollinated fruits)	5	2.39	0.043
	Fruit yield per plant (Non-pollinated fruits)	5	0.63	0.681
	Weight of fruit	5	1.64	0.157
	Number of fruit	5	2.49	0.036
	Percentage fruit set	5	2.62	0.029
	Days of fruit growing	5	1.59	0.173
	Diameter of fruit	5	0.76	0.580
	Length of fruit	5	1.15	0.339
	Total number of seeds per plant	5	6.78	0.000
	Number of seeds per fruit	5	6.53	0.000
Nutrients and Pollination	Fruit yield per plant (Pollinated fruits)	5	4.09	0.002
	Fruit yield per plant (Non-pollinated fruits)	5	0.30	0.915
	Weight of fruit	5	1.40	0.232
	Number of fruit	5	1.32	0.262
	Percentage fruit set	5	0.91	0.476
	Days of fruit growing	5	1.40	0.235
	Diameter of fruit	5	2.99	0.016
	Length of fruit	5	2.12	0.072
	Total number of seeds per plant	5	1.30	0.564
	Number of seeds per fruit	5	0.78	0.270

* df means Degree Freedom. F is the statistical value of ANOVA.

**Table 3:** Correlation analysis of cucumber flower, fruit, and seed traits using Pearson’s linear simple correlation with two-tailed *p*-values.

		Female Flowers Per Plant	Number Fruits Per Plant	Non-Pollinated Female Flowers	Male Flowers Per Plant	Non-Pollinated Male Flowers	Fruit Yield Per Plant	Total Seeds Per plant
Number fruits per plant	r	0.337						
	p	0.000						
	n	117						
Non-pollinated female flowers	r	0.349	0.049					
	p	0.000	0.601					
	n	117	117					
Male flowers per plant	r	0.451	0.088	0.246				
	p	0.000	0.345	0.007				
	n	117	117	117				
Non-pollinated male flowers	r	0.259	−0.173	0.147	0.444			
	p	0.005	0.063	0.113	0.000			
	n	117	117	117	117			
Fruit yield per plant	r	0.337	.892	−0.053	0.135	−0.152		
	p	0.000	0.000	0.569	0.145	0.101		
	n	117	117	117	117	117		
Total seeds per plant	r	0.236	0.721	−0.033	0.139	−0.146	0.849	
	p	0.01	0.000	0.725	0.136	0.116	0.000	
	n	117	117	117	117	117	117	
Shoot mass	r	0.387	−0.039	0.380	0.293	0.523	−0.065	−0.067
	p	0.000	0.681	0.000	0.001	0.000	0.488	0.473
	n	117	117	117	117	117	117	117
Seeds per fruit	r	0.051	−0.034	0.135	0.124	−0.007	−0.057	−0.042
	p	0.593	0.714	0.146	0.182	0.942	0.539	0.656
	n	117	117	117	117	117	117	117
Fruit weight	r	0.019	0.077	0.093	0.101	−0.051	0.053	0.012
	p	0.861	0.465	0.379	0.336	0.629	0.614	0.911
	n	92	92	92	92	92	92	92

## Data Availability

Data are contained within the article.

## References

[1] Zhang D.Y., Zhang D.Y. (2003). Reproductive Ecology of Plants. Plant Life-History Evolution and Reproductive Ecology.

[2] Zhang T., Tan D.Y. (2008). Adaptive significances of sexual system in andromonoecious *Capparis spinosa* (*Capparaceae*). J. Syst. Evol..

[3] Buonaiuto D.M., Wolkovich E.M. (2021). Differences between flower and leaf phenological responses to environmental variation drive shifts in spring phenological sequences of temperate woody plants. J. Ecol..

[4] Creux N.M., Brown E.A., Garner A.G., Saeed S., Scher C.L., Holalu S.V., Yang D.I., Maloof J.N., Blackman B.K., Harmer S.L. (2021). Flower orientation influences floral temperature, pollinator visits and plant fitness. New Phytol..

[5] Strauss S.Y. (1997). Floral characters link herbivores, pollinators, and plant fitness. Ecology.

[6] Rusman Q., Lucas-Barbosa D., Hassan K., Poelman E.H. (2020). Plant ontogeny determines strength and associated plant fitness consequences of plant-mediated interactions between herbivores and flower visitors. J. Ecol..

[7] Ansaldi B.H., Weber J.J., Franks S.J. (2018). The role of phenotypic plasticity and pollination environment in the cleistogamous, mixed mating breeding system of *Triodanis perfoliata*. Plant Biol..

[8] Li X.M., She D.Y., Zhang D.Y., Liao W.J. (2015). Life history trait differentiation and local adaptation in invasive populations of *Ambrosia artemisiifolia* in China. Oecologia.

[9] Bell G. (1985). On the function of flowers. Proc. R. Soc. Lond. B..

[10] Caruso C.M. (2004). The quantitative genetics of floral trait variation in Lobelia: Potential constraints on adaptive evolution. Evolution.

[11] Worley A.C., Barrett S.C.H. (2000). Evolution of floral display in *Eichhornia paniculata* (*Pontederiaceae*): Direct and correlated responses to selection on flower size and number. Evolution.

[12] Sargent R.D., Goodwillie C., Kalisz S., Rees R.H. (2007). Phylogenetic evidence for a flower size and number trade-off. Am. J. Bot..

[13] Atlan A., Hornoy B., Delerue F., Gonzalez M., Pierre J.S., Tarayre M. (2015). Phenotypic plasticity in reproductive traits of the perennial shrub ulex europaeus in response to shading: A multi-year monitoring of cultivated clones. PLoS ONE.

[14] Sun S., Zhang D.Y., Ives A.R., Li Q.J. (2011). Why do stigmas move in a flexistylous plant?. J. Evol. Biol..

[15] Perry L.E., Dorken M.E. (2011). The Evolution of Males: Support for Predictions from Sex Allocation Theory Using Mating Arrays of *Sagittaria latifolia* (*Alismataceae*). Evolution.

[16] Torices R., Mendez M. (2011). Influence of inflorescence size on sexual expression and female reproductive success in a monoecious species. Plant Biol..

[17] Carper A.L., Adler L.S., Irwin R.E. (2016). Effects of florivory on plant-pollinator interactions: Implications for male and female components of plant reproduction. Am. J. Bot..

[18] Peruzzi L., Mancuso E., Gargano D. (2012). Males are cheaper, or the extreme consequence of size/age-dependent sex allocation: Sexist gender diphasy in *Fritillaria montana* (*Liliaceae*). Bot. J. Linn. Soc..

[19] Burd M. (1994). Bateman Principle and Plant Reproduction—The Role of Pollen Limitation in Fruit and Seed Set. Bot. Rev..

[20] Christopher D.A., Mitchell R.J., Karron J.D. (2020). Pollination intensity and paternity in flowering plants. Ann. Bot..

[21] Xu G.H., Wolf S., Kafkafi U. (2001). Interactive effect of nutrient concentration and container volume on flowering, fruiting, and nutrient uptake of sweet pepper. J. Plant Nutr..

[22] Garcia M.B. (2003). Sex allocation in a long-lived monocarpic plant. Plant Biol..

[23] Wright J.W., Meagher T.R. (2004). Selection on floral characters in natural Spanish populations of Silene latifolia. J. Evol. Biol..

[24] Ganeshaiah K.N., Shaanker R.U. (1988). Seed abortion in wind-dispersed pods of dalbergia-sissoo—Maternal regulation or sibling rivalry. Oecologia.

[25] Marcelis L.F.M., Heuvelink E., Hofman-Eijer L.R.B., Den Bakker J., Xue L.B. (2004). Flower and fruit abortion in sweet pepper in relation to source and sink strength. J. Exp. Bot..

[26] Rosenheim J.A., Alon U., Shinar G. (2010). Evolutionary balancing of fitness-limiting factors. Am. Nat..

[27] Schreiber S.J., Rosenheim J.A., Williams N.W., Harder L.D. (2015). Evolutionary and ecological consequences of multiscale variation in pollen receipt for seed production. Am. Nat..

[28] Rodriguez-Garcia E., Olano J.M., Leroux O., Mezquida E.T. (2019). Deciphering the role of reproductive investment, pollination success and predispersal seed predation on reproductive output in *Juniperus thurifera*. Plant Ecol. Divers.

[29] Song Y.P., Ma K.F., Ci D., Chen Q.Q., Tian J.X., Zhang D.Q. (2013). Sexual dimorphic floral development in dioecious plants revealed by transcriptome, phytohormone, and DNA methylation analysis in Populus tomentosa. Plant Mol. Biol..

[30] Barber N.A., Adler L.S., Bernardo H.L. (2011). Effects of above- and belowground herbivory on growth, pollination, and reproduction in cucumber. Oecologia.

[31] Bai S.N., Xu Z.H. (2013). Unisexual cucumber flowers, sex and sex differentiation. Int. Rev. Cel. Mol. Bio..

[32] Boualem A., Troadec C., Camps C., Lemhemdi A., Morin H., Sari M.A., Fraenkel-Zagouri R., Kovalski I., Dogimont C., Perl-Treves R. (2015). A cucurbit androecy gene reveals how unisexual flowers develop and dioecy emerges. Science.

[33] Liu W.F., Qin Z.W., Xin M., Zhou X.Y., Yang J., Wang C.H. (2018). Analysis of CsPAP-fib regulation of cucumber female differentiation in response to low night temperature conditions. Sci. Hortic..

[34] Nicodemo D., Malheiros E.B., De Jong D., Couto R.H.N. (2013). Enhanced production of parthenocarpic cucumbers pollinated with stingless bees and Africanized honey bees in greenhouses. Semin. Cienc. Agrar..

[35] Zhou Y., Ahammed G.J., Wang Q., Wu C.Q., Wan C.P., Yang Y.X. (2018). Transcriptomic insights into the blue light-induced female floral sex expression in cucumber (*Cucumis sativus* L.). Sci. Rep..

[36] Diola V., Orth A.I., Guerra M.P. (2008). Reproductive biology in monoecious and gynoecious cucumber cultivars as a result of IBA application. Hortic. Bras..

[37] Dorken M.E., Barrett S.C.H. (2003). Gender plasticity in *Sagittaria sagittifolia* (*Alismataceae*), a monoecious aquatic species. Plant Syst. Evol..

[38] Golenberg E.M., West N.W. (2013). Hormonal interactions and gene regulation can link monoecy and environmental plasticity to the evolution of dioecy in plants. Am. J. Bot..

[39] Paquin V., Aarssen L.W. (2004). Allometric gender allocation in *Ambrosia artemisiifolia* (*Asteraceae*) has adaptive plasticity. Am. J. Bot..

[40] Van Drunen W.E., Dorken M.E. (2012). Trade-offs between clonal and sexual reproduction in *Sagittaria latifolia* (*Alismataceae*) scale up to affect the fitness of entire clones. New Phytol.

[41] Halpern S.L., Adler L.S., Wink M. (2010). Leaf herbivory and drought stress affect floral attractive and defensive traits in Nicotiana quadrivalvis. Oecologia.

[42] Groeneveld J.H., Tscharntke T., Moser G., Clough Y. (2010). Experimental evidence for stronger cacao yield limitation by pollination than by plant resources. Perspect. Plant Ecol..

[43] Motzke I., Tscharntke T., Wanger T.C., Klein A.M. (2015). Pollination mitigates cucumber yield gaps more than pesticide and fertilizer use in tropical smallholder gardens. J. Appl. Ecol..

[44] Chen M., Zuo X.A. (2018). Pollen limitation and resource limitation affect the reproductive success of *Medicago sativa* L. BMC Ecol..

[45] Jacome-Flores M.E., Delibes M., Wiegand T., Fedriani J.M. (2018). Spatio-temporal arrangement of Chamaerops humilis inflorescences and occupancy patterns by its nursery pollinator, *Derelomus chamaeropsis*. Ann. Bot..

[46] Vallejo-Marin M., Rausher M.D. (2007). The role of male flowers in andromonoecious species: Energetic costs and siring success in *Solanum carolinense* L. Evolution.

[47] Ramsey M., Vaughton G. (2001). Sex expression and sexual dimorphism in subdioecious *Wurmbea dioica* (*Colchicaceae*). Int. J. Plant Sci..

[48] Rocheta M., Sobral R., Magalhaes J., Amorim M.I., Ribeiro T., Pinheiro M., Egas C., Morais-Cecilio L., Costa M.M.R. (2014). Comparative transcriptonnic analysis of male and female flowers of monoecious *Quercus suber*. Front Plant Sci..

[49] Teixido A.L., Valladares F. (2014). Pollinator-mediated phenotypic selection does not always modulate flower size and number in the large-flowered Mediterranean shrub *Cistus ladanifer* (*Cistaceae*). Bot. J. Linn. Soc..

[50] Dittmar P.J., Monks D.W., Schultheis J.R. (2009). Maximum potential vegetative and floral production and fruit characteristics of watermelon pollenizers. Hortscience.

[51] Low J.E., Aslund M.L.W., Rutter A., Zeeb B.A. (2011). The effects of pruning and nodal adventitious roots on polychlorinated biphenyl uptake by *Cucurbita pepo* grown in field conditions. Environ. Pollut..

[52] Choi E.Y., Cho I.H., Moon J.H., Woo Y.H. (2012). Impact of secondary-lateral branch removal during watermelon production. Hortic Environ. Biotechnol..

[53] Nabizadeh E., Taherifard E., Gerami F. (2011). Effect of pruning lateral branches on four varieties of medicinal castor bean plant (*Ricinuscommunis* L.) yield, growth and development. J. Med. Plants Res..

[54] Lord J.M., Westoby M. (2012). Accessory costs of seed production and the evolution of angiosperms. Evolution.

[55] Marsal J., Basile B., Solari L., DeJong T.M. (2003). Influence of branch autonomy on fruit, scaffold, trunk and root growth during Stage III of peach fruit development. Tree Physiol..

[56] Hu Y.C., Song Z.W., Lu W.L., Poschenrieder C., Schmidhalter U. (2012). Current soil nutrient status of intensively managed greenhouses. Pedosphere.

[57] Moreno D.A., Pulgar G., Ruiz J.M., Villora G., Romero L. (1998). Optimum nutrient range in cucumber (*Cucumis sativus* L. cv. Brunex F-1 plants): I. Nitrogen and phosphorus parameters. Phyton Int. J. Exp. Bot..

[58] Baker D.N., Kanekal S.G., Li X., Monk S.P., Goldstein J., Burch J.L. (2004). An extreme distortion of the Van Allen belt arising from the ‘Hallowe’en’ solar storm in 2003. Nature.

[59] Dinesh M.R., Rekha A., Ravishankar K., Praveen K.S., Santosh L.C. (2007). Breaking the intergeneric crossing barrier in papaya using sucrose treatment. Sci. Hortic..

[60] Owuor B.O., Owino F. (1993). Control pollination and pollen management in *Sebania sesban* (L). Merr. Euphytica.

[61] Dalkilic Z., Mestav H.O. (2011). In vitro pollen quantity, viability and germination tests in quince. Afr. J. Biotechnol..

[62] Godini A. (1981). Counting pollen grains of some almond cultivars by means of haemocytometer. Rivista della Ortoflorofrutticoltura Italiana.

[63] Burd M. (2004). Offspring quality in relation to excess flowers in *Pultenaea gunnii* (*Fabaceae*). Evolution.

[64] Sutherland S. (1986). Patterns of fruit-set—What controls fruit-flower ratios in plants. Evolution.

[65] Gepts P. (2004). Domestication as a long-term selection experiment. Plant Breed. Rev..

